# Editorial: Meningioma: From Basic Research to Clinical Translational Study

**DOI:** 10.3389/fonc.2021.750690

**Published:** 2021-10-22

**Authors:** Allen Ho, Hailiang Tang

**Affiliations:** ^1^ Department of Neurosurgery, Stanford University School of Medicine, San Francisco, CA, United States; ^2^ Department of Neurosurgery, Huashan Hospital, Fudan University, Shanghai, China

**Keywords:** meningioma, basic research, clinical translational study, adjuvant radiotherapy, targeted chemotherapy

Meningioma is thought to originate from arachnoidal cells in the central nervous system (CNS) and accounts for approximately 30% of all brain tumors (1). Most meningiomas are low grade benign brain tumors (such as fibroblastic meningioma and meningothelial meningioma), which belong to the World Health Organization (WHO) grade I and have a good prognosis after surgery. About 20% of meningiomas are high grade malignant brain tumors and belong to the WHO grade II (such as atypical meningioma) or WHO grade III (such as anaplastic meningioma) (2). Malignant meningiomas, sometimes may evolve from benign meningiomas, are more aggressive. They have an increased risk of recurrence after surgery and significant mortality rates. Currently, surgical resection combined with adjuvant radiotherapy is the main treatment strategy for malignant meningiomas, and no effective targeted chemotherapies have been developed (3–5).

Thus, in this Research Topic (https://www.frontiersin.org/research-topics/12083/meningioma-from-basic-research-to-clinical-translational-study), we collected more than 60 manuscripts discussing meningioma issues involving from basic research to clinical translational study, intend to deeply state those unresolved problems in meningioma. For example, its recurrence factors, how to improve the prognosis for malignant meningioma patients, what about the targeted chemotherapy for refractory meningioma?

## Clinical Aspects of Meningioma

Meningiomas are diverse in intracranial locations and pathology, which are classified into three WHO grades and 15 histological subtypes. Sometimes meningiomas presented preferred intracranial locations, which may reflect potential biological features. In this meningioma issue collection, Sun et al. analyzed the preferred locations of meningioma according to different biological characteristics. Malignant meningiomas, compared to benign meningiomas, are more aggressive and have higher risk of recurrence after surgery. Clinical prognosis of meningioma patient is closely related to the WHO grades: patients with benign meningiomas have 5-year survival rates of 92%; however, the 5-year survival rates decrease to 78% in atypical meningiomas, and drop to 47% in anaplastic meningiomas. Currently, effective treatment for malignant meningiomas is still difficult (6). Here, we reviewed several manuscripts discussing meningioma treatment.

Surgical resection of anterior clinoidal meningiomas remains challenge because of its complicated relationships with surrounding tissues (internal carotid arteries and optic nerves). Xu et al. found that the meningeal structures around the anterior clinoid process may guide and determine the origin and extension of anterior clinoidal meningiomas.

Matthias Schneider et al. analyzed 32 patients with spheno-orbital meningiomas underwent surgical treatment to evaluate the recovery of tumor-associated proptosis. They found that the exophthalmos index could provide a comparable standard in the evaluation.

Currently, consensus is limited regarding the optimal transcranial approaches for surgical resection of olfactory groove meningioma. Feng et al. used meta-analysis to review operative and peri-operative outcomes of unilateral compared with bilateral approaches for such kind of meningioma.

Skull base meningiomas with extracranial extensions are rarely reported. Liu et al. described the clinical features, surgical management and clinical outcomes of these meningiomas and investigated risk factors associated with progression free survival (PFS).

Bu et al. introduced their surgical experience of 162 patients with small cerebellopontine angle meningioma, and showed that surgical treatment should be the first choice for these meningiomas.

Sylvian fissure meningiomas are relatively rare and have different characteristics compared with typical meningiomas. Cai et al. reported such kind of meningioma in their paper.

Wang et al. discussed clear cell meningioma (CCM), a very rare subtype of meningioma. They concluded that CCM patients have a favorable survival rate. Patients diagnosed at 21-60 years old and patients with spinal CCMs have a better prognosis.

Yang et al. in their paper, proved that prophylactic antiepileptic drugs (AEDs) treatment for 186 patients with supratentorial meningioma from their center, does not reduce the rate of perioperative seizures.

Interestingly, Li et al. showed that elderly meningioma patients might present significantly polarization trend in maintaining long-term independence after surgery.

Intracranial hemangiopericytoma and meningioma are both meningeal neoplasms, but they have extremely different malignancy and outcomes. Because of their similar radiological characteristics, they are difficult to distinguish before surgery, leading to a high rate of misdiagnosis. Wei et al. showed that the proposed Meningioma Diagnostic Tool could assist in preoperative diagnosis to distinguish hemangiopericytoma from meningioma.

## Adjuvant Radiotherapy for Meningioma

Currently, surgical resection combined with adjuvant radiotherapy is still the main treatment options for malignant meningiomas, for instance anaplastic meningioma. And so far, no effective targeted chemotherapies have been implemented. However, adjuvant radiotherapy is controversy in some circumstances, for example, in the cases of atypical meningiomas.

For atypical meningiomas, the combination of gross total resection (GTR) and adjuvant radiotherapy is still a controversial therapeutic strategy to improve prognosis. Zhang et al. analyzed the factors influencing the prognosis on atypical meningiomas patients treated with GTR and adjuvant radiotherapy in their paper.

He et al. performed a meta-analysis study of effectiveness of postoperative adjuvant radiotherapy in atypical meningioma patients after gross total resection (GTR), and showed that postoperative adjuvant radiotherapy could improve the 5-year local control rate and 5-year PFS (progression free survival).

Wang et al. in their study created a new “prognostic score” that allows personalized recommendations for post-operative adjuvant radiotherapy in patients with high grade meningioma.

## Risk Factors for Recurrence in Meningioma

Here, we also reviewed some manuscripts focusing on meningioma recurrence, discussing the risk factors. For instance, Zhu et al. retrospectively studied 392 meningioma patients after surgery to identify the independent risk factors of recurrence, and constructed a scoring system for the prediction of the risk of postoperative recurrence.

Alexander Fadi Haddad et al. proved that subtotal resection (STR), posterior fossa location, nuclear atypia, and elevated MIB-1 index are prognostic factors for WHO grade I meningioma recurrence. Moreover, MIB-1 index >4.5% is prognostic for recurrence in patients with gross total resection (GTR).

Zhang et al. discussed malignant progression in atypical meningiomas, and they showed that malignant progression was significantly correlated with an increased incidence of recurrence in gross total resection (GTR) plus early EBRT (external beam radiotherapy)-treated intracranial atypical meningiomas.

Wu Ye et al. investigated the relationship between clinicopathological characteristics of atypical meningiomas (AM) and its post-operative recurrence.

Neutrophil-to-lymphocyte ratio (NLR) has been reported as a prognostic factor for several solid tumors. However, the prognostic value of NLR in meningiomas is lack. Yuki Kuranari et al. reported that NLR may be a cost-effective and novel preoperatively usable biomarker in patients with meningiomas. Besides, Chen et al. also confirmed the correlation and clinical significance of preoperative fibrinogen and neutrophil-lymphocyte ratio (F-NLR) scoring system with 3-year PFS (progression-free survival) of patients with atypical meningioma.

## Updated Reviews of Meningioma

Several reviews in this meningioma issue, discussing advanced management of meningioma from current therapy strategies, novel therapeutic approaches, and future directions, are worth reading. For instance, Taylor Anne Wilson et al. reviewed the update on management of atypical and anaplastic meningiomas, and discussed the risk factors, classification, and molecular biology of meningiomas as well. Besides, Zhao et al. systematically introduced the current treatment progress of meningioma in their review paper entitled with “An overview of managements in meningiomas”. Moreover, Kristin Huntoon et al. reviewed clinicopathological and molecular aspects in meningioma. While there are currently no good adjuvant chemotherapeutic agents available, recent advances in the genomic and epigenomic landscape of meningiomas are being explored for potential targeted therapy for meningioma (7, 8). Shao et al. reviewed advances in chromosomal variations and molecular mechanisms involved in the progression of meningioma, and highlighted the association with malignant biological behavior including cell proliferation, angiogenesis, increased invasiveness, and inhibition of apoptosis.

## Basic Researches on Meningioma

In this meningioma topic collection, we also recruited several manuscripts focused on meningioma basic studies from aspects of receptors, sex hormones, and meningioma cells level. Those researches are thought to be promisingly paving ways for future targeted therapies for meningiomas.

Despite high recurrence rate of atypical and malignant subtypes, there is no approved drug investigated for meningioma. Maya Hrachova et al. evaluated efficacy and safety of Sandostatin LAR (octreotide, a kind of somatostatin analogs) in patients with progressive, and/or recurrent meningioma, and identified subset of patients who were more likely to benefit from this treatment. Wu et al. also discussed the clinical significances of somatostatin receptor (SSTR)-2 in meningioma, a G-protein-coupled receptor and can be activated by somatostatin or its synthetic analogs.

Female sex hormones may influence meningioma development. Francesco Maiuri et al. proved that the biological behavior of meningiomas and their pathological findings, including progesterone receptor (PR) expression, are not correlated with the different hormone related conditions in premenopausal female patients. Contraceptives and fertilization therapies should be avoided in patients with meningiomas.

Accumulating evidence indicated that long non-coding RNA maternally expressed gene 3 (MEG3) participated in the progression of meningioma. However, the potential mechanisms of MEG3 need further investigation. Ding et al. showed that MEG3 mediated the aggressive behaviors of meningioma cells *via* miR-29c/AKAP12 axis, supporting that MEG3 served as a promising biomarker for the diagnosis and treatment of human meningioma. Zhang et al. also studied the effect of microRNA-221/222 radiosensitivity in meningiomas in their paper.

## Other Aspects of Meningioma Research

Currently, multiple methods have been applied for meningioma research, including genomics, proteomics, epigenetics, radiomics, multi-omics, etc. These techniques will help in deeply explore those aggressive meningiomas.

Ma et al. performed genome-wide genotyping for cranial meningiomas in 383 Chinese patients and identified 9,821 copy number variations, showing patients with diverse clinical features had distinct tumor copy number variations profiles.

The DNA methylation-based meningioma classification published in 2017 (9) used DNA copy number analysis, mutation profiling, and RNA sequencing to distinguish six clinically relevant methylation classes, which contributed to a better prediction of meningioma recurrence and prognosis. Shen et al. summarized the key findings of recent studies on the methylation status and genetic mutations of meningioma and discussed the current deficits of WHO grading.

Studies have shown mitochondrial genome (mtDNA) content varies in many malignancies. However, its distribution and prognostic values in high-grade meningioma remain largely unknown. In the retrospective study, Hua et al. assessed a putative correlation between the mtDNA content and clinical characteristics. They found that high mtDNA content was associated with better outcome in WHO grade III meningioma.

Gu et al. reviewed the latest advancements of radiomics and its applications in the prediction of the pathological grade, pathological subtype, recurrence possibility, and differential diagnosis of meningiomas.

Growing evidence demonstrated the potential of multi-omics study (including genomics, transcriptomics, epigenomics, proteomics) for meningiomas diagnosis and mechanistic links to underlying therapeutic targets. In the review paper, Liu et al. provided a timely and necessary study of such scientific basis for further treatment of meningiomas.

## Conclusions

In conclusion, as discussed above, all of the recent developments are creating new prospects for effective molecularly driven diagnosis, classification and therapy of meningiomas (9–11). However, there is still a long way to go in the study of meningioma from many aspects. There are still many problems, including its cell origin (12), to be solved for this very complicated brain tumor. Here, we had just discussed a very little knowledge on meningioma in this meningioma topic collection. However, with continued research on the mechanisms of meningioma pathogenesis, the screening and development of new drug targets are forthcoming.

## Author Contributions

All authors listed have made a substantial, direct, and intellectual contribution to the work and approved it for publication.

## Funding

This work was supported by grant (No. 19140900105) from the Shanghai Science and Technology Commission.

## Conflict of Interest

The authors declare that the research was conducted in the absence of any commercial or financial relationships that could be construed as a potential conflict of interest.

## Publisher’s Note

All claims expressed in this article are solely those of the authors and do not necessarily represent those of their affiliated organizations, or those of the publisher, the editors and the reviewers. Any product that may be evaluated in this article, or claim that may be made by its manufacturer, is not guaranteed or endorsed by the publisher.
